# Congenital Knee Dislocation: A Rare Orthopedic Enigma in Rural Nepal

**DOI:** 10.7759/cureus.95361

**Published:** 2025-10-24

**Authors:** Rupak Kandel, Asmita Khanal, Anita Dahal, Bibek Panthee

**Affiliations:** 1 Department of Orthopedic Surgery, Karnali Care International Hospital and Research Center, Surkhet, NPL; 2 Department of Public Health, Manmohan Memorial Institute of Health Sciences, Kathmandu, NPL; 3 Department of Pediatrics, Karnali Provincial Hospital, Surkhet, NPL; 4 Department of Emergency Medicine, Nepal Police Hospital, Surkhet, NPL

**Keywords:** congenital dislocation of the knee, orthopedic opd cases, orthopedic procedures, pediatric case, public healthcare

## Abstract

Congenital knee dislocation (CKD) (genu recurvatum congenitum) is a rare neonatal orthopedic deformity characterized by hyperextension of the knee joint and potentially associated with intrauterine mechanical factors, quadriceps contracture, or syndromic disorders. Early recognition is essential, as prompt conservative management can prevent long-term disability and reduce the need for surgical intervention. The objective of this single case report is to describe a rare presentation of bilateral congenital knee dislocation managed successfully through early conservative treatment in a rural and resource-limited setting of Nepal, highlighting the timeline of improvement and long-term outcome.

We report a full-term female neonate born via institutional delivery in a remote region of Dailekh District, Nepal, who presented within 24 hours of birth with bilateral knee hyperextension of approximately 100°, consistent with type II congenital knee dislocation (anterior tibial subluxation without complete dislocation). The neonate was treated with gentle manipulation and serial above-knee plaster casting, with casts changed every 7-10 days to gradually increase knee flexion from 0° to 30°, while the toes were left exposed for monitoring of circulation and swelling due to the remote location of the patient’s family. Noticeable improvement was observed after three casting sessions over a four-week period, and by six weeks, both knees achieved near-neutral to mildly flexed resting positions, demonstrating improved alignment and stability. Follow-up radiographs at six weeks, six months, two years, and four years confirmed maintained reduction, normal knee alignment, intact growth plates, and age-appropriate motor milestones without pain, instability, or functional limitations. This case highlights that early diagnosis and conservative management of congenital knee dislocation can achieve excellent long-term anatomical and functional outcomes, even in resource-limited settings and during challenging circumstances, emphasizing the importance of continuous follow-up and parental counseling to ensure adherence and awareness in managing rare congenital orthopedic conditions.

## Introduction

Congenital knee dislocation (CKD), often referred to as genu recurvatum congenitum, is a concerning and surprising discovery at birth. In the 1960s, it was agreed that the term congenital knee dislocation, which is defined by the forward displacement of the proximal tibia on the femoral condyles, encompassed all hyperextended knees present at birth [1]. It is a rare condition, occurring in approximately one in 100,000 live births, with an incidence of around 1% of congenital hip dislocation. It is more commonly seen in females than in males [1,2]. The diagnosis is typically made at birth through clinical observation of abnormal hyperextension of the knee joint [3].

The exact etiology and pathophysiology of congenital knee dislocation (genu recurvatum) remain unclear; however, several contributing factors have been proposed. These include intrauterine mechanical influences such as oligohydramnios, uterine abnormalities, and fetal malpositioning, particularly breech presentation, as well as maternal trauma, limited intrauterine space, quadriceps contracture or fibrosis, absence of the suprapatellar pouch, and hypoplasia or deficiency of the anterior cruciate ligament [4,5]. Histological investigations have revealed fibrotic alterations in the anterior joint capsule and quadriceps tendon, corroborating the idea that soft tissue disease plays a role in joint malalignment [6].

Clinically, congenital knee dislocation usually manifests at birth as a stiff, hyperextended knee that is resistant to passive flexion. It is divided into three classes according to its severity: grade I is hyperextension without subluxation, grade II is anterior tibial subluxation, and grade III is total dislocation. Early identification is essential since conservative therapy, such as periodic casting and gentle manipulation, can usually produce great results when started promptly. Surgical procedures, such as femoral osteotomy, anterior capsule release, or quadriceps lengthening, may be required if conservative therapy is delayed or fails [7-10]. Therefore, it is essential to screen affected patients for associated anomalies.

Through this case report, we aim to raise awareness among clinicians about bilateral congenital knee dislocation, a rare congenital condition, and to strengthen their confidence in counseling parents regarding treatment options and long-term outcomes. This four-year follow-up case is particularly significant because it was managed successfully in a rural, resource-limited setting where access to specialized orthopedic care and advanced imaging is scarce. It demonstrates that early diagnosis and timely conservative management can yield excellent functional recovery and long-term stability, even in challenging healthcare environments, thereby addressing a gap in existing literature on outcomes from such settings.

## Case presentation

This case involved a full-term neonate diagnosed with type II congenital knee dislocation, presenting with severe bilateral knee hyperextension at birth. The case was identified in Dailekh District, a remote region of Nepal. The neonate received early conservative management, including gentle manipulation and serial above-knee casting.

**Figure 1:**
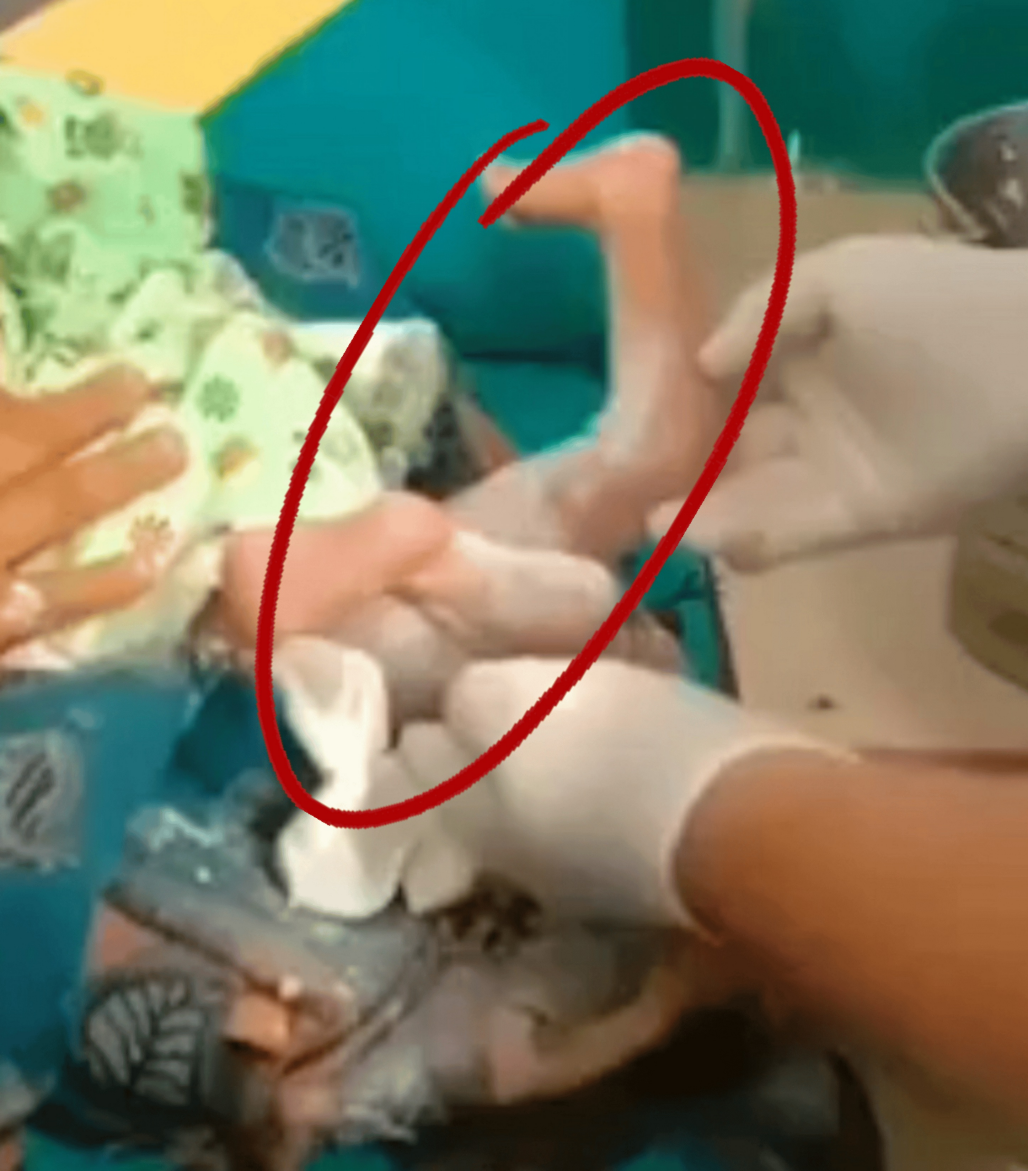
Clinical image showing congenital knee dislocation at birth

A full-term female neonate was delivered via spontaneous vaginal delivery, with the mother having received regular antenatal care and an uneventful delivery. At birth, the neonate transitioned well with no systemic complications; however, physical examination revealed severe bilateral knee hyperextension of approximately 100°, consistent with type II congenital knee dislocation (Figure 1).

**Figure 2:**
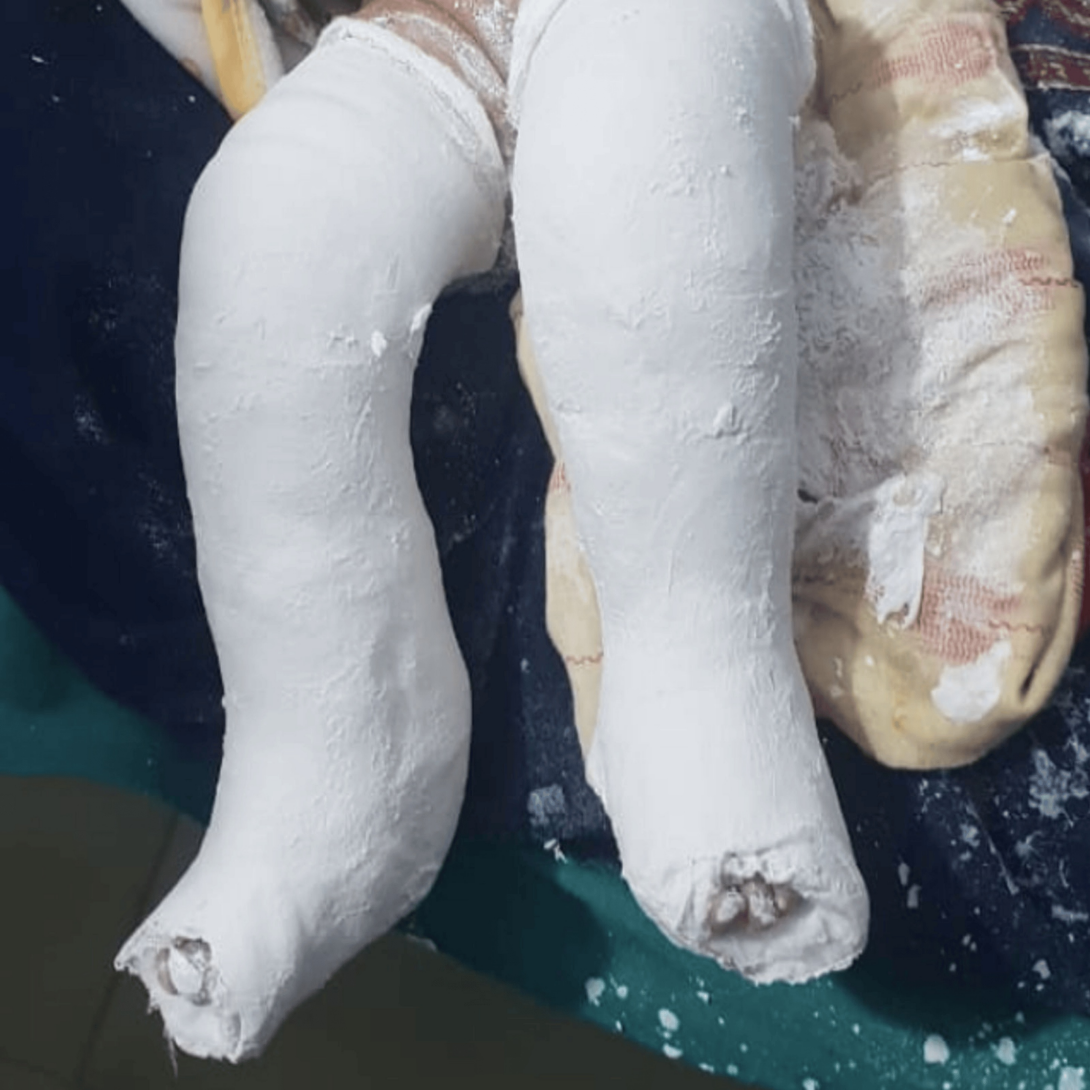
Post-reduction casting in bilateral congenital knee dislocation

Initial gentle manipulation achieved partial reduction, but sustained flexion could not be maintained, indicating persistent joint instability. Early conservative management was initiated at a regional medical facility under orthopedic supervision, using serial above-knee plaster casting with casts changed at regular intervals to gradually increase knee flexion from 0° to 30°, while the toes were left exposed to monitor circulation and swelling. This approach was selected due to the remote location of the patient’s family, which limited access to frequent hospital follow-up (Figure 2).

**Figure 3:**
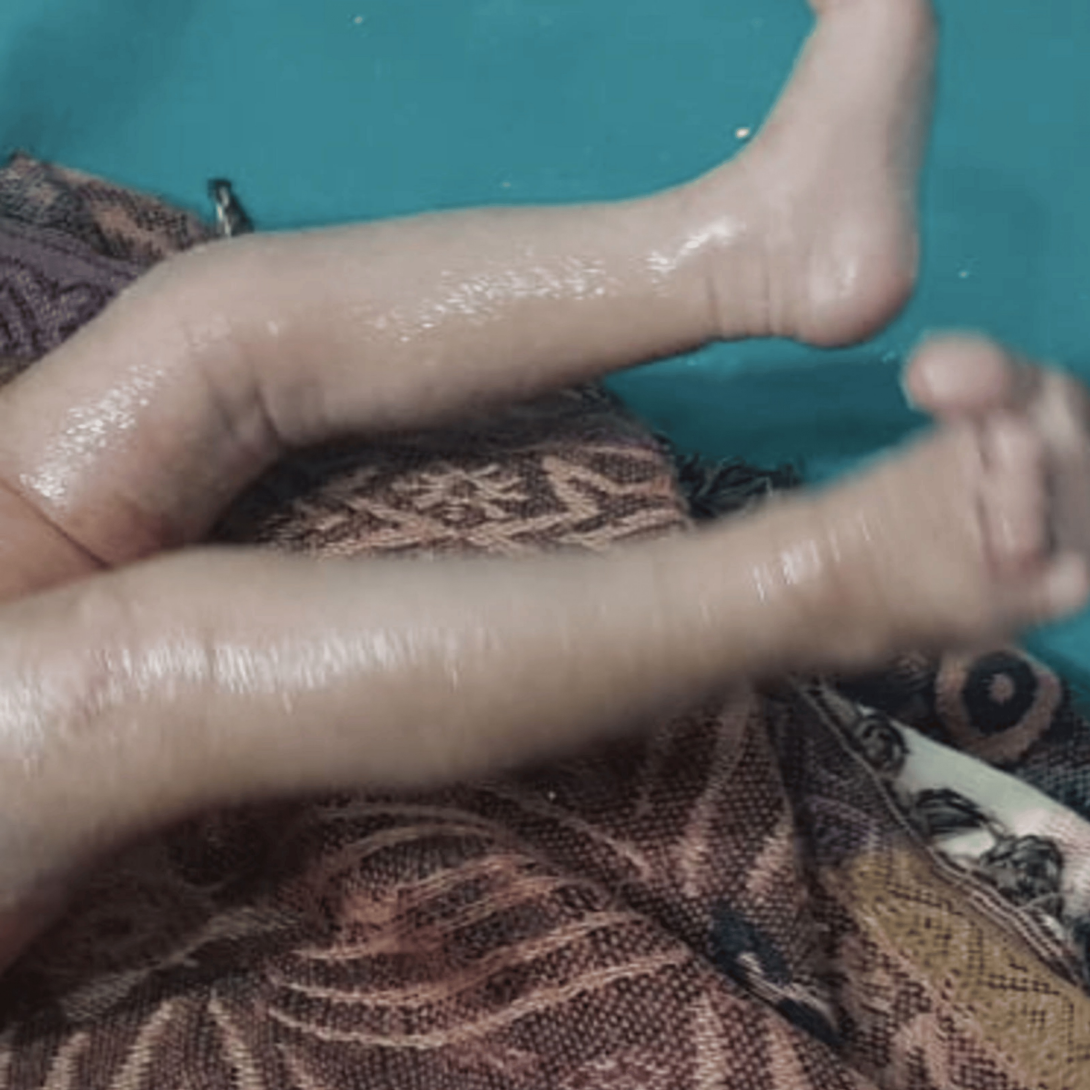
Lower limbs of the neonate following plaster cast removal for type II bilateral congenital knee dislocation

Over the course of the treatment, the hyperextension deformity improved markedly, and at cast removal, both knees rested in a near-neutral to mildly flexed position, demonstrating improved alignment and stability, with intact skin and no pressure sores or other complications. The feet maintained normal alignment, confirming the absence of secondary deformities (Figure 3).

**Figure 4:**
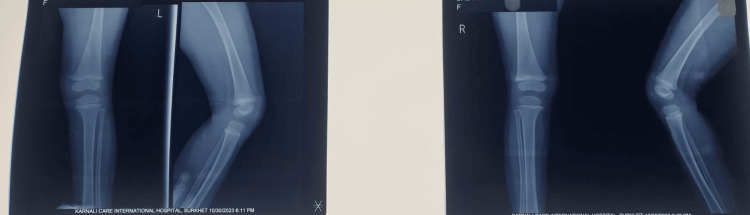
Anteroposterior and lateral radiographs of the right and left knee at two years of age

Follow-up radiographs obtained on March 10, 2023, approximately one and a half years after the initial assessment on October 14, 2021, demonstrated well-aligned femorotibial articulations with preserved bony morphology and joint spaces, without subluxation, deformity, or growth plate abnormalities, confirming excellent medium-term outcomes (Figure 4).

**Figure 5:**
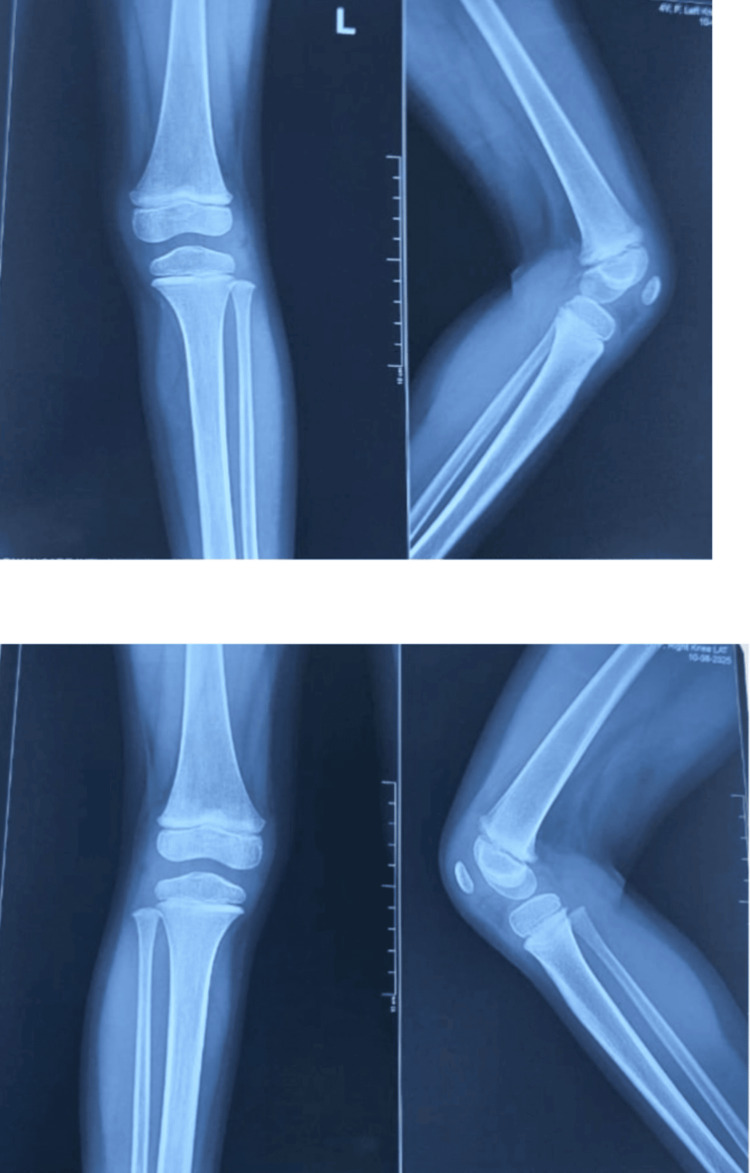
Follow-up radiographs at four years showing well-aligned knees with normal joint congruency and intact growth plates

Subsequent radiographs, including the last one obtained in August 2025, approximately four years after the initial case on October 14, 2021, demonstrated stable knee alignment, normal joint congruency, and intact epiphyseal plates, with no evidence of recurrent dislocation, deformity, or degenerative changes, confirming long-term joint stability (Figure 5).

**Figure 6:**
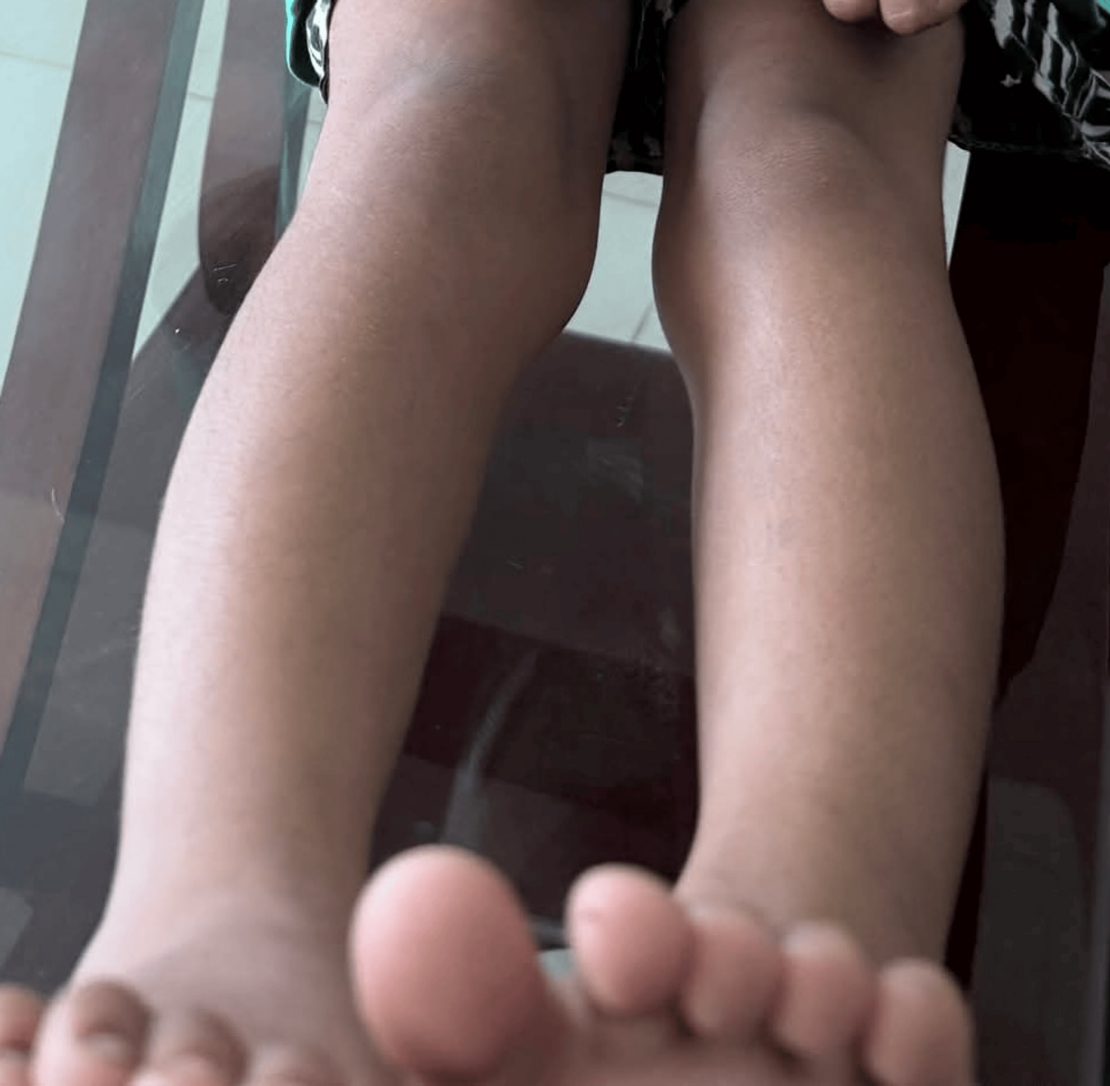
Clinical appearance of the child’s lower limbs at follow-up, showing well-aligned knees in a normal position

At the most recent clinical evaluation, the child’s lower limbs appeared normal, with proper knee alignment and no residual hyperextension or deformity, demonstrating complete recovery and highlighting the efficacy of early diagnosis, gentle manipulation, and serial casting in achieving excellent long-term outcomes for congenital knee dislocation, even in resource-limited settings (Figure 6).

This four-year follow-up report demonstrates the effectiveness of early conservative management in achieving complete anatomical and functional recovery in a resource-limited setting during the COVID-19 pandemic. Serial casting improved knee flexion from 0° to 30° within six weeks. No syndromic or systemic abnormalities were detected, and growth and developmental assessments confirmed normal milestone attainment. Comparative pre- and post-treatment details have been incorporated into the figures for greater clarity and clinical relevance.

## Discussion

Congenital knee dislocation (CKD) is a rare limb malformation, usually noticeable at birth. Initial clinical evaluation should focus on assessing the extent of knee hyperextension, identifying anterior skin creases, and determining the reducibility of the dislocation. These characteristics are crucial for guiding treatment decisions and predicting outcomes [1]. Plain radiographs, including lateral and anteroposterior views of the knee, remain the preferred diagnostic modality. They help confirm the diagnosis by showing anterior displacement of the proximal tibia relative to the femoral condyles and can detect associated fractures or bony abnormalities [2].

CKD may present as an isolated condition or as part of a broader set of skeletal abnormalities. Mechanical (extrinsic) factors contributing to CKD include congenital absence of the cruciate ligaments, quadriceps fibrosis, or intrauterine positional issues such as prolonged extended breech positioning [3]. CKD may also be associated with intrinsic or genetic disorders, including Larsen syndrome, Desbuquois syndrome, Collins-Pope syndrome, Marfan syndrome, Down syndrome, Ehlers-Danlos syndrome, and Golgi-resident 3'-phosphoadenosine-5'-phosphate phosphatase (GPAPP) deficiency [4-6]. In this case, maternal and neonatal evaluations did not reveal any syndromic or genetic abnormalities, suggesting a mechanical etiology likely related to intrauterine positional factors.

In the present case, early diagnosis facilitated prompt initiation of conservative management through gentle manipulation and serial casting. This approach resulted in excellent functional and radiological outcomes, supporting existing literature that early intervention can often prevent the need for surgical correction [7,8]. Although initial radiographs were lost due to technical issues, follow-up clinical examinations and imaging at two and four years demonstrated normal femorotibial alignment with no evidence of recurrent dislocation, joint instability, or growth plate abnormality. After treatment, the child began walking at the normal developmental age of one year and, by four years, was able to walk and run independently without assistance, achieving age-appropriate gross motor milestones and remaining asymptomatic.

This case reinforces that early recognition and conservative management of CKD can achieve optimal outcomes, even in resource-limited settings. Continuous follow-up and community education are essential to ensure adherence to treatment protocols and to dispel misconceptions regarding the management of rare congenital conditions [9,10].

## Conclusions

Congenital knee dislocation (CKD) is a rare but correctable neonatal orthopedic condition. Early diagnosis and prompt initiation of conservative treatment, such as gentle manipulation and serial casting, can result in excellent functional and radiological outcomes, even in resource-limited settings. In this single case, timely intervention allowed the child to achieve normal growth and development without long-term complications, avoiding the need for surgical correction. However, these findings may not be generalizable to all CKD cases, particularly those associated with syndromic or severe presentations.

Equally important is the parental experience, which highlights the emotional impact of such a diagnosis. The initial anxiety and uncertainty gradually transformed into relief and confidence as treatment progressed successfully. Over time, the positive outcome not only reassured the family but also helped raise awareness within the community that rare congenital conditions are treatable with appropriate care. This underscores the need for early recognition, parental counseling, and community education in managing rare congenital deformities such as CKD.
